# Factors Impacting Clinicians’ Adoption of a Clinical Photo Documentation App and its Implications for Clinical Workflows and Quality of Care: Qualitative Case Study

**DOI:** 10.2196/20203

**Published:** 2020-09-23

**Authors:** Christine Jacob, Antonio Sanchez-Vazquez, Chris Ivory

**Affiliations:** 1 Anglia Ruskin University Cambridge United Kingdom; 2 University of Applied Sciences Northwestern Switzerland Brugg Switzerland; 3 Innovation and Management Practice Research Centre Anglia Ruskin University Cambridge United Kingdom

**Keywords:** mHealth, mobile health, telehealth, eHealth, health tech, digital health, user-engagement, dermatology, wound care, mobile phone

## Abstract

**Background:**

Mobile health (mHealth) tools have shown promise in clinical photo and wound documentation for their potential to improve workflows, expand access to care, and improve the quality of patient care. However, some barriers to adoption persist.

**Objective:**

This study aims to understand the social, organizational, and technical factors affecting clinicians’ adoption of a clinical photo documentation mHealth app and its implications for clinical workflows and quality of care.

**Methods:**

A qualitative case study of a clinical photo and wound documentation app called imitoCam was conducted. The data were collected through 20 in-depth interviews with mHealth providers, clinicians, and medical informatics experts from 8 clinics and hospitals in Switzerland and Germany.

**Results:**

According to the study participants, the use of mHealth in clinical photo and wound documentation provides numerous benefits such as time-saving and efficacy, better patient safety and quality of care, enhanced data security and validation, and better accessibility. The clinical workflow may also improve when the app is a good fit, resulting in better collaboration and transparency, streamlined daily work, clinician empowerment, and improved quality of care. The findings included important factors that may contribute to or hinder adoption. Factors may be related to the material nature of the tool, such as the perceived usefulness, ease of use, interoperability, cost, or security of the app, or social aspects such as personal experience, attitudes, awareness, or culture. Organizational and policy barriers include the available clinical practice infrastructure, workload and resources, the complexity of decision making, training, and ambiguity or lack of regulations. User engagement in the development and implementation process is a vital contributor to the successful adoption of mHealth apps.

**Conclusions:**

The promising potential of mHealth in clinical photo and wound documentation is clear and may enhance clinical workflow and quality of care; however, the factors affecting adoption go beyond the technical features of the tool itself to embrace significant social and organizational elements. Technology providers, clinicians, and decision makers should work together to carefully address any barriers to improve adoption and harness the potential of these tools.

## Introduction

### Background

Mobile health (mHealth) tools are gaining importance in health care as they show promise in several capacities, ranging from efficiencies and time-saving [1,2] to decreasing clinicians’ workload and enhancing access to care [3,4]. The use of health apps has also contributed to tackling patients’ information needs and making them feel more empowered [5]. The data generated by such tools also help clinicians adapt and customize treatment plans accordingly [6], improving patients’ quality of care via personalized treatments [5,6]. Although research shows that clinicians have an overall positive attitude toward mHealth, some barriers to adoption persist [7,8].

In clinical photo documentation and dermatology, research has shown the potential of mHealth tools in managing and preventing skin issues [9-11] and that clinicians and patients recognize their value and are generally willing to use them [12-14].

The Global Observatory of eHealth in the World Health Organization defines mHealth as “medical and public health practice supported by mobile devices, such as mobile phones, patient monitoring devices, personal digital assistants (PDAs), and other wireless devices” [15]. mHealth solutions differ from other information and communication technology apps in the sense that they are typically user-driven, accessible, and affordable [16]; consequently, it is very important to understand better the factors affecting user adoption and the respective implications for workflow and quality of care.

Therefore, this research focuses on understanding the factors affecting clinicians’ adoption of mHealth and its implications for clinical practice through a case study of a clinical photo and wound documentation app called imitoCam and its adoption by clinicians in Switzerland and Germany.

Founded in 2016 in Switzerland, imito AG is a clinical photo and wound documentation start-up offering the imitoCam app for medical photo and video documentation and wound measurement. It also supports system interoperability and electronic medical record (EMR) integration. Visuals from the app are presented in Multimedia Appendix 1. The app’s key features are explained in Multimedia Appendix 2 and include secure photo documentation; direct patient identification via barcode; measurement of the area (and length, width, and circumference) of wounds and specimens; patient timelines to better understand the case progression; categorization of images to enable photo search; and team collaboration via a chat function, for example, second opinions. The visual in Multimedia Appendix 3 demonstrates seamless integration between the app and existing hospital systems. The wide adoption of the app in 15 hospitals and clinics across Switzerland and Germany at the time of writing this paper made it an ideal candidate to explore mHealth usage and its implications for clinical workflow and quality of patient care.

### Objective

This work is a part of a larger study focusing on understanding clinicians’ adoption of mHealth; a previous study published earlier presented a more detailed account of our theoretical approach [2]. In this paper, we employ a sociotechnical framework [17] to build a comprehensive analysis of the factors affecting clinicians’ adoption of mHealth and its implications for workflow and clinical practice following these 3 main steps:

- Investigating the material aspects of technology and their limitations by identifying the utility and limitations of the app as perceived by the users
- Connecting the material aspects of technology to the tasks it enables and facilitates by highlighting the real constraints to its potential as seen by the user
- Identifying the processes resulting from these affordances and determining the resulting interactions taking place in the organization by identifying the implications for clinical practice and quality of care

The following section explains the research method and how the interview questions and subsequently, the analysis, stemmed from these 3 steps.

## Methods

A qualitative paradigm was implemented as it gives priority to “the voices of participants” and the individual and unique “reflexivity of the researcher” [18] and for the rich insights it provides, which can help understand clinicians’ individual perceptions in different ways, which cannot be achieved by quantitative methods [19,20].

### Data Collection

Data were collected via in-depth, semistructured interviews that were conducted via Skype, Google Hangout, or telecon. Physical artifacts such as screenshots of the app, the devices it can be used on, and examples of user feedback were collected to develop a broader assessment of the studied app [21]. Data collection took place from July 2019 to January 2020, and a total of 20 interviews were conducted with 18 participants working in 8 clinics and hospitals across Switzerland and Germany (2 interviews were preparatory alignment interviews about the tool’s features and capabilities). The interviews were conducted via telecon and lasted between 17 min and 90 min, with a median of 35 min. A total of 4 participants sent their responses electronically via email as they did not have the time for a live call. Interviews were conducted and recorded by the first author (CJ) in English. The interview topic guide is available in Multimedia Appendix 4. The research themes and questions were developed in line with the Methodological Guidelines for the Study of Materiality and Affordances by Leonardi to crystallize the focus on the data collected in the interviews [17]. Accordingly, the themes in the interview guide were clustered into 3 categories, starting with an understanding of the tool’s utilities and limitations, followed by investigating the technical and social factors affecting adoption and a discussion about organizational and policy factors and implications. The data collection phase continued until an acceptable level of saturation was reached, which was when new data did not generate new insights anymore [20].

### Sampling Techniques and Participant Profiles

We used a purposive sample where participants were chosen based on their ability to specify rich and in-depth information about the app and its usage [18,19]. Key informants in imito AG were contacted, and snowball sampling was consequently used to identify suitable participants in partner hospitals and clinics. The key selection criteria were that participants must be clinicians or medical informatics experts in one of the partner hospitals or clinics using the app and must have experienced the app for at least several months. The medical informatics experts had a very good overview of the app and its features, given their access to usage statistics, user feedback, and their constant engagement with clinicians to ensure its successful implementation and sustainability. To minimize the risk of selection bias that might result from the key informants selectively picking users with a positive predisposition toward the app, it was decided that the participants would be asked if they could, in turn, suggest other colleagues who used the app and were willing to participate.

The participants worked in 8 hospitals and clinics across Switzerland and Germany, and the sample consisted of 9 clinicians (one of them was also an imito AG team member), 5 medical informatics experts, and 4 other members from the imito AG team, as shown in Table 1.

**Table 1:** Sample demographics and characteristics (N=18).

Demographics		Values
**Function, n (%)**		
	Clinicians	9 (50)^a^
	Medical informatics experts	5 (28)
	Other imito AG team members	4 (22)
**Gender, n (%)**		
	Female	3 (17)
	Male	15 (83)
Technological awareness (on a scale of 1-10), mean (SD)		7.5 (2.3)
Health care experience (years), mean (SD)		13.4 (10.4)
mHealth experience (years), mean (SD)		3.9 (2.2)
Location		Switzerland and Germany

^a^One of them is also an imito AG team member.

### Data Analysis and Ethical Considerations

Thematic analysis was used to identify and extract the relevant themes and interpret their potential meaning and interrelationships among [19,22]. Computer-assisted qualitative data analysis software, QSR’s NVivo was used for data coding. Excerpts were chosen to create an account that expressed the narrative of each theme in a way that helped the reader better understand the analysis. The first author (CJ) conducted the interviews and performed the initial analysis and coding; she is a digital strategist with more than 18 years of experience and has contributed to the creation and realization of several digital tools in health care. The second author (ASV) reviewed the coding; any cases of disagreement were discussed in conjunction with the last author (CI) and mutually agreed upon. The phases of the thematic analysis are clarified in detail in Multimedia Appendix 5 [22].

Our themes were mostly influenced by sociotechnical theory and Leonardi’s methodological guidance [17] looking into the technical, social, and organizational factors interacting with shape technology adoption. We also took into account the emerging themes in the most used frameworks for studying mHealth adoption based on a systematic review that we had published earlier [23]. Accordingly, our themes were also influenced by other prominent frameworks such as the Technology Acceptance Model [24], the Diffusion of Innovation theory [25], different forms of extensions of the Unified Theory of Acceptance and Use of Technology [26-29], and the Consolidated Framework for Implementation Research [30,31]. Multimedia Appendix 6 clarifies in detail how each of our themes was influenced by one or more of the existing frameworks and the themes that emerged from the data.

Ethical approval was obtained from the faculty research ethics panel under the terms of Anglia Ruskin University’s Research Ethics Policy. All participants were briefed about the research background and signed a consent form agreeing to participate.

## Results

### Accounting for the Materials: Utility and Limitations

**Figure 1:**
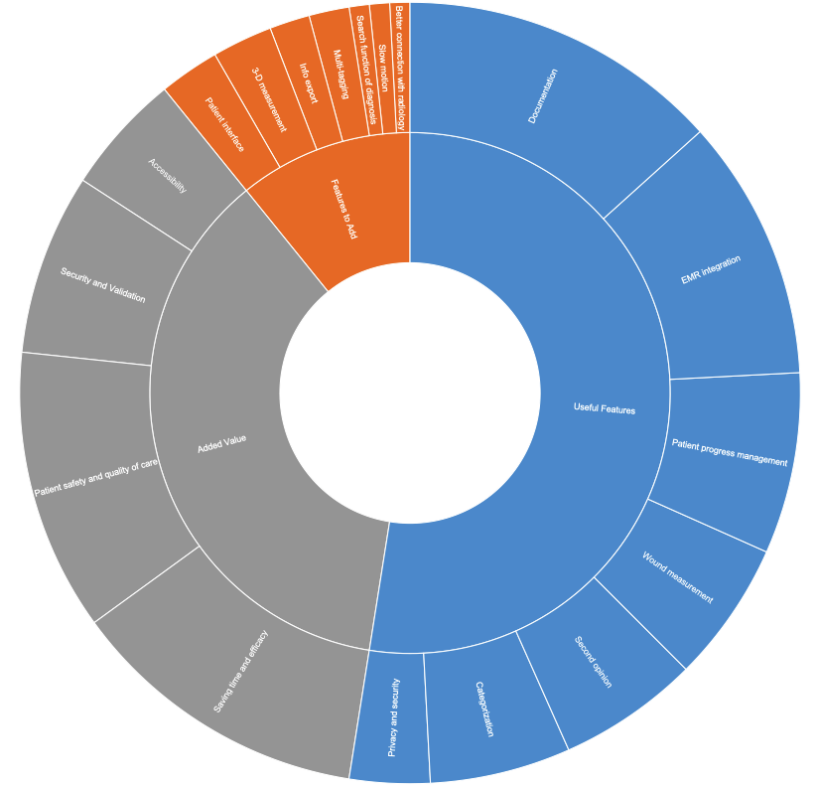
Utility and limitations of the app.

As a first step, we investigated the app’s utility and limitations by exploring the most used features, the perceived added value, and the potential limitations or ideas for improvement. Figure 1 shows the themes in these 3 categories and their respective subthemes, reflecting the frequency of each theme (frequencies reflect the number of participants that mentioned that specific theme).

Participants were first asked to name the app features that they used the most to better understand the technological artifacts that they found most useful. Photo and wound documentation (n=16) was the most used feature, followed by EMR integration (n=13), which enables clinicians to link the photos to the right patient in the hospital information system. This was followed by patient progress management (n=9), which gives them visibility of each patient’s development over time; wound measurement (n=7) using the imito calibration markers (quick response [QR] codes) for image calibration; and the possibility of obtaining a second opinion (n=7) through the chat function. Participants equally valued the categorization and classification feature (n=7) that enables them to tag the photos and easily find them later. Finally, they also appreciated the privacy and security (n=4) that the app offers as it ensures compliance with the General Data Protection Regulation (GDPR).

Participants were then asked to explain how the app helped them and their patients daily to better understand imito’s utility from their perspective. Saving time and efficacy (n=15) was clearly an added value for most of the participants. They also saw an improvement in patient safety and quality of care (n=14) as well as data security and validation (n=9) and explained that the mobility of the app improves data accessibility through the compact overview that it offers (n=6).

To complete the picture, the participants were asked about any limitations they faced or features they would like to add to the app. Limitations mentioned by the participants included the absence of a patient interface, lack of offline functionality, data quality of the user-defined hashtags (eg, typos in user-defined hashtags can limit their searchability), possibility of accessing the imito data from the EMR but not the other way around (eg, the user cannot see the patient data stored in the EMR from the imito tool on their mobile device unless they were captured using the app), the necessity of using physical calibration markers (QR codes) for the measurement function can be cumbersome when the users do not have them on hand, and that the app can only be used inside the hospital as it requires connection to the hospital’s system. Participants also expressed their desire for some additional features such as a patient interface (n=3), 3-dimensional measurement (n=3), export of information from the app via email (n=2), multitagging of body parts or regions to cover injuries involving different body parts (n=2), a search function for diagnoses (n=1), enhancement of the slow-motion video function (n=1), and a better connection with radiology devices (n=1).

The key themes and subthemes, their frequencies, and some sample quotes about utility and limitations are summarized in Table 2 for clarity.

**Table 2:** Most useful features, added value, and features to add.

Theme		Sample quotes	
**Most useful features**			
	Photo and wound documentation (n=16)		“So that was what we were searching for. A product which is possible to make good photo documentation and the option that it can connect to the system here in the hospital and so we have the picture in the medical file of the patient. And this is the main feature why we use imito because it was the first system that makes it possible in a fast way” [C^a^11]
	Electronic medical record integration (n=13)		“By scanning the name of the patient or his patient identification number, the document is linked to the hospital information system and the photos are stored in the patient file” [C18]
	Patient progress management (n=9)		“very good visibility of the development of each individual case” [P^b^6] “So, it’s easier to follow the progress of healing” [I^c^12] “Current photos can be immediately compared with older recordings so one can assess the progress of wound healing” [C18]
	Wound measurement (n=7)		“And one further, very good benefit...you can place QR codes in the photo. They are like sticky notes, and you can place it on the screen next to it, and this is referencing it in terms of size. So...you can measure width, length, and even the surface area of a wound. You can decide if a certain area is becoming smaller or larger or whatever” [C5]
	Second opinion (n=7)		“Networking with other authorized users is the next step and enables interdisciplinary communication” [C18]
	Categorization and classification (n=7)		“And before you upload a choice of videos or photos, you are asked to tag your photos by selecting a body region from an illustrated human. And furthermore, you can add hashtags such as ‘burn wound’” [C5]
	Privacy and security (General Data Protection Regulation compliance; n=4)		“the only thing today is to send a picture...to other people to get second opinion is using WhatsApp... And it would make my life easier if we would have some good solutions which you are allowed to use, then we could forbid the rest” [I10]
**Added value**			
	Saving time and efficacy (n=15)		“My expectation was to improve the documentation and make more photos per visit than before, and that certainly worked” [C16]
	Patient safety and quality of care (n=14)		“So, it’s an objective parameter, and you see it’s getting better. When it’s getting better, you continue. If you see it’s stagnant, it remains, or it gets bigger, this helps quickly to detect that your medical measures are not good. And then instead of treating the patients another four weeks or three months, you change. You take action and reflect and you change” [C3] “You can show the patient how his progress is going on and the picture can say more than a lot of words. It’s just useful for everyone who’s using it” [I12] “there’s also quality benefits that we can directly compare with the initial status” [C16]
	Security and validation (n=9)		“And after the upload, no data is left on the device itself, in the gallery, for example. So, this app sends the images to the hospital’s database and there it is as safe as the hospital database can be, and this is the really strong benefit” [C5] “So, it’s not any more than that you have patient pictures that are just flying around somewhere and have no names on it, and you can’t map them back to the patient, which was also a matter of patient security and safety” [I13]
	Accessibility and compact overview (n=6)		“It’s providing the relevant data at the right time and the right context” [P1] “Sometimes we need a dermatologist. And so, we can call them. And this is what we want for the whole hospital, that every station is using this for the documentation so that we can sit here in the front of my PC and have a look...So, I don’t have to run over there, make the picture, run back or get everything I have, for example, with me. And so, it makes it, for me, easier” [C11]
**Features to add**			
	Patient interface (n=3)		“So as soon as the electronic health record comes about, then it should be possible to push all that information into the electronic health record of the patients. So, it will be more and more important to let the patient participate on that process” [I13]
	3-dimensional measurement (n=3)		“The depth of the wound could also be measured by imito. There was a system that had a laser. And with the laser, you had also the depth of the wound” [C3]
	Information export (n=2)		“And probably, also...the house doctor, and whoever can use the app as an information tool...we have to take the pictures, and then you have to put them in some order, and then you have to export it as a PDF or whatever. That would be great if that could also be mobile and flexible” [C14] “Once taken, the pictures are imprisoned in imito. You cannot send a GP an email with the photo...So, you are just losing time all the time. You can’t reuse the pictures from within imito mobile for presentations because we don’t get them out. Whereas respectively, you have to do screenshots. You have to cut the screenshots. You have to send via mail” [C3]
	Multitagging body parts (n=2)		“So right now, you can just choose one body part. And to choose two body parts, this would be an important thing, I think because sometimes we have injuries which are going—they are bigger or just going over different body parts” [I12]
	Search function of diagnosis (n=1)		“I’m missing, namely the long list of diagnoses...and a search function for the diagnosis” [C16]
	Better slow-motion video (n=1)		“it would be beneficial to have a better slow-motion feature in the videos. And I know tools for coaches, for example, golf coaches, and what they can do is while playing in slow motion, they can stop and then they can measure angles, for example” [C5]
	Better connection with radiology devices (n=1)		“we would like to also allow a better connection between those radiological devices...and providing them safe and secure authentication of patients” [CP8]

^a^C: clinician.

^b^P: provider.

^c^I: informatics.

### Accounting for Materiality: Constraints and Affordances

**Figure 2:**
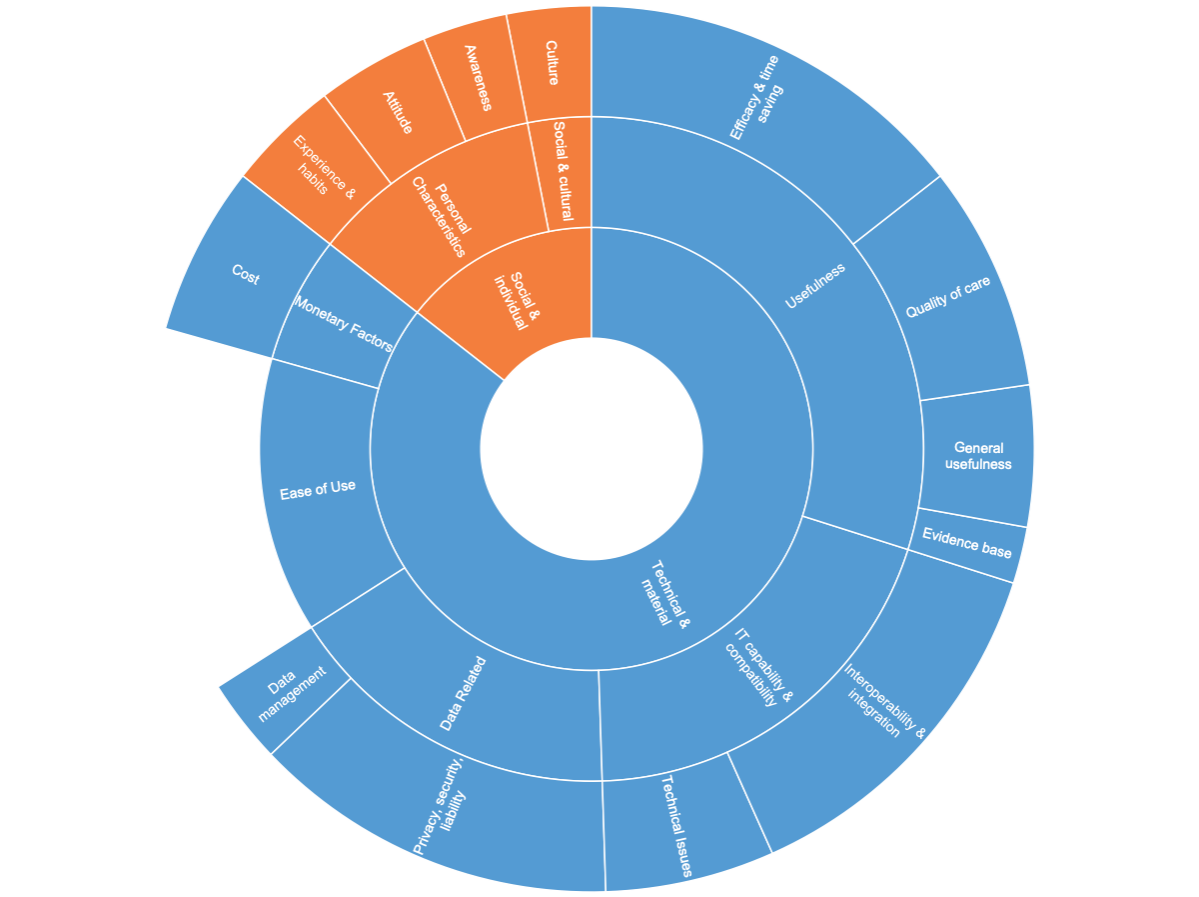
Technical and social factors impacting user adoption.

We then examined the app’s materiality by looking into the constraints and affordances affecting the tool’s adoption from technical and social perspectives. Figure 2 shows the themes in these 2 categories and their respective subthemes, reflecting the frequency of each of them.

Technical and material factors evolved around 5 key themes: usefulness, information technology (IT) capability and compatibility, data-related factors, ease of use, and monetary factors. Usefulness is clearly crucial for adoption; most participants explained that the efficacy of the app and the time saved encouraged adoption (n=14), improvements in the quality of patient care also resulted in more usage of the app (n=8), its usefulness in general was valuable (n=5), and the role that it could play in generating scientific evidence via better documentation was also considered (n=2).

The IT capability and integration factors were focused on the app’s interoperability (n=13), showing how important it was for the users that the chosen app could integrate effectively with the hospital’s local information system, so as to avoid double work and documentation errors, aside from technical issues (n=6) such as poor connectivity, log-in difficulties, or short battery life that are mostly perceived as barriers to adoption. Although data-related factors mostly focused on privacy, security, and data liability issues related to the use of mHealth tools and sharing patient data (n=13), such issues can be overcome with secure and GDPR-compliant tools such as the studied app as it ensures patient data security and privacy; and the challenges related to data management (n=3), especially with the myriad of data generated by such tools which makes the resulting amount of data hard to manage.

Ease of use (n=13) was mostly perceived as a facilitator in the case of imito, with several users mentioning that an easy-to-use tool was central to adoption. Furthermore, monetary factors such as the cost entailed by these tools (n=6) may play a role, not only with regard to the apps’ licensing costs but also the related system integration and infrastructure costs. These key technical factors and subthemes, their frequencies, and some sample participant quotes about each of them are summarized in Table 3 for clarity.

Factors affecting adoption went beyond the technical aspects to also cover some social and cultural elements. The users’ personal characteristics such as previous experience and habits (n=4), attitude toward technology and change (n=4), and their awareness of the value of such tools (n=3) may also play a role in their decision to adopt such an app. In addition, cultural factors (n=3), such as other people’s views and perceptions of using mobile devices at the workplace, may also play a role in the adoption decision. These key social factors and subthemes, their frequencies, and some key participant quotes about each of them are summarized in Table 4.

**Table 3:** Technical factors as expressed by the participants.

Theme		Sample quotes	
**Usefulness**			
	Efficacy and time-saving (n=14)		“It created efficiency. Before it (photo documentation) took maybe three, four, five minutes, and now it takes 30 seconds” [I^a^9] “So, it is a lot of time-savings and quality improvements” [P^b^15]
	Quality of care (n=8)		“(the case progression overview) helps to quickly detect if your medical measures are not good. And then instead of treating the patients (with the same treatment) another four weeks or three months, you change. You take action and reflect and you change” [C^c^3] “you take photographs, and you see what are the changes over months or not...(these photos) save time for very specific descriptions that you otherwise place in your report” [C5] “In the operating room, the photos are not available. So, the clinician has to either just have a good guess what happened in his memory or get to retrieve the photo somewhere else. (With the app) there is really benefits in the treatments because you have the things available when you need them” [P15]
	General usefulness (n=5)		“The clear benefit for the clinical routine” [P1] “The aspect of creating new possibilities that didn’t exist before” [P15]
	Evidence base (n=2)		“I think the more people use the app, we have to see whether it’s good for statistics or identifying relevant cases in terms of research” [C5] “We also expected benefits in terms of scientific studies, simplification of treatment algorithms, and networking of inpatient and outpatient treatment pathways” [C18]
**IT^d^ capability and compatibility**			
	Interoperability and integration (n=13)		“But there are barriers, mainly the IT integration requirement” [P1] “You can access the app via any mobile device, and logging on with your personal hospital account is possible. The app is then linked to the hospital’s database and allows to identify patients by entering their personal details or to scan a barcode and this will give you the patient” [C5] “The EMR integration in this regard is a challenge both from a cost perspective and the support availability perspective” [I13] “(the app) is much easier than taking an individual camera as it’s directly available within the patient file which is very useful” [C16]
	Technical issues (n=6)		“of course, an app like this needs a lot of battery. So, we have to load the battery two or three times a day” [C11] “And sometimes, but this is not a problem of the imito app, it’s a problem of the system here, when we have no Wi-Fi, it gets more difficult to make a documentation and to save it” [C16] “I was too frustrated with the log-in process and now that we have the possibility to log in with face ID, it has proved to be a marvel” [C16]
**Data-related factors**			
	Privacy, security, and liability (n=13)		“The limitations and problems are rather in the legal area, as the sending of sensitive patient data is very restrictive in Germany. Legal and technical requirements for secure data transfer must be dealt with. Good photo documentation supports the sociomedical and legal issues” [C18] “And altogether you just have to still follow the hospital rules about data security and all that stuff, so that’s also an adoption factor. The data security” [I12]
	Data management (n=3)		“And the other thing we noticed is that it needs some kind of controlling in the future because it is so well accepted that some users overdo it. And we are not limited in terms of data capacity, storage space” [C5] “We have more pictures in this time we roll out the devices. So, I don’t know if it’s always good to have just more content, if it’s also in the right context and is it useful and that stuff. But we have more” [I9]
Ease of use (n=13)		“I would say the process has to be very easy. So, when you want to have an app like this, it has to be easy, fast, and secure” [I9] “It’s very important to have an easy self-explanatory tool for nurses to use. Otherwise, they won’t do it, understandably” [C14]	
Monetary factors (cost; n=6)		“We have a cost in this technological interoperability” [CP8] “Barriers for establishing such tools are the investment costs, eg, set up of a secure WLAN, equipment, and licensing cost” [C18]	

^a^P: provider.

^b^I: informatics.

^a^C: clinician.

^d^IT: information technology.

**Table 4:** Social factors as expressed by the participants.

Theme		Sample quotes
**Personal characteristics**		
	Experience and habits (n=4)	“the medical field, as well, has a new generation now, getting to work more with digital health like a tablet or a smartphone” [C^a^11] “And then the head of the dialysis found out that she really had people on her staff that didn’t have a smartphone. But I think it’s not the general population in this ward” [C14]
	Attitude (n=4)	“And now with electronic health record opening all of it come these changes that can be challenging for physicians that were not used to that or that are resistant to changes” [CP^b^8]
	Awareness (n=3)	“It’s more of an awareness and training topic than functionality...to take a picture, that’s very easy, you are used from your own cell phone. But if you make a wound measurement, okay, how does it work? And the QR code and—you have to have some information about this” [I^c^9]
Social and cultural factors (n=3)		“Maybe on this point of view that, if you ever have a phone in your hands, many people think, ‘Okay. You are gaming something, or you are on social media.’ But this is a working device. And we are in a change now that the patients—they see, ‘Okay. I can do something with the doctor’” [I9]

^a^C: clinician.

^b^P: provider.

^b^I: informatics.

### Accounting for Materialization: Organizational Factors and Their Implications

**Figure 3:**
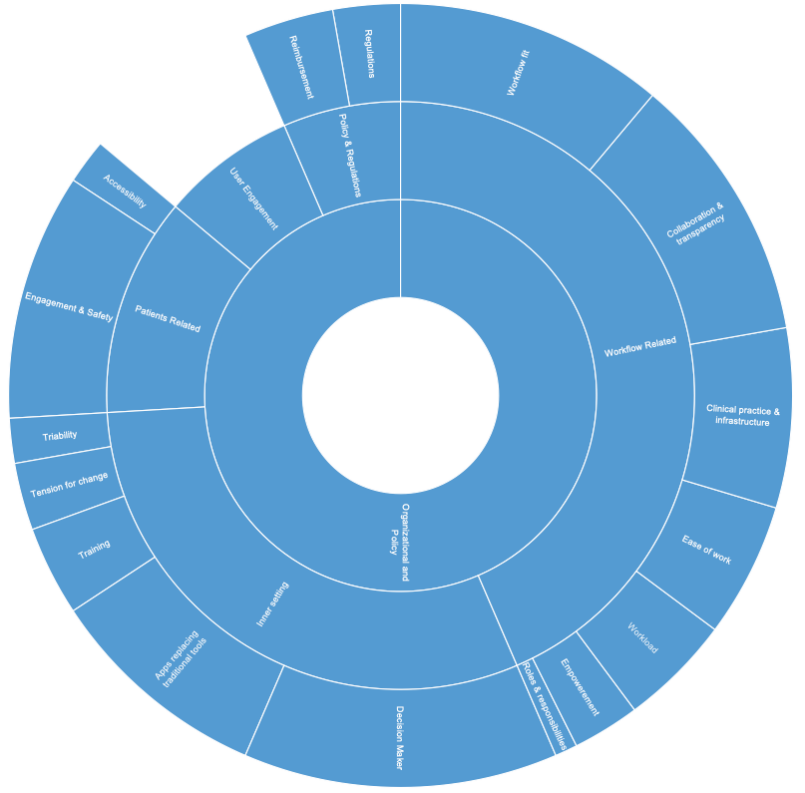
Organizational and policy factors impacting user adoption.

The organizational factors and their implications for clinical workflow and quality of care were then discussed in detail. Figure 3 shows the themes in this category and their respective subthemes, reflecting the frequency of each theme.

Organizational and policy factors revolved around 5 key themes: workflow-related themes, organization’s specific inner setting, patient-related factors, user engagement, and policy and regulations. Workflow-related themes were mostly focused on workflow fit and location flexibility (n=12), showing that the app’s fit with clinicians’ existing work practices encouraged its adoption. Improvements in collaboration and transparency may also increase usage (n=12), whereas traditional clinical practices and infrastructure, such as the lack of use of mobile devices in hospitals, could pose a challenge (n=8). Users naturally favor apps that make their daily work easier (n=6), although existing high workload or lack of resources could sometimes be a barrier (n=5). Some participants explained that the data availability and accessibility facilitated by the app empowered them on the job (n=3). However, apps could also alter some existing roles and responsibilities (n=1). Apps such as imitoCam, for example, have the potential to reduce or even eliminate the role of a professional photographer at the hospital.

Factors related to the organizational inner setting include the complex nature of the decision-making process in hospitals (n=14). Slow decision making can slow down the adoption decision or even prevent it. Participants also pinpointed as to how mHealth tools are replacing traditional tools such as digital cameras in the case of imito (n=10). The importance of training and education to facilitate usage was also highlighted (n=4). The organization’s propensity for innovation and appetite for change may also play a role in the adoption decision (n=3), for example, when the organization desires to be perceived as innovative to better compete with other hospitals and clinics. The possibility of trying and piloting the new tool may also facilitate adoption as it minimizes the risk of a full rollout until users have tried the app (n=2).

Patient-related factors were also central and focused mainly on how the app might impact patient engagement and safety (n=11) and enhance access to care (n=2). Many participants also emphasized the significance of user engagement in the development process (n=8), and several imito team members acknowledged the importance of this factor in the success of the tool, explaining that they realized the importance of onboarding their app into the hospital’s system and workflows, rather than the other way around.

Policy and regulations in general (n=3), and reimbursement and funding (n=4) in particular, were equally perceived as essential factors for adoption. These key organizational and policy factors, their subthemes, frequencies, and some representative participant quotes are summarized in Table 5.

**Table 5:** Organizational and policy factors as expressed by the participants.

Theme		Sample quotes
**Workflow-related theme**		
	Workflow fit and location flexibility (n=12)	“before, you had to go onto the station, take the camera. Now, you have it in your pocket right next to you. You can log in with the face ID, take a picture and send it” [I^a^9] “it’s (the app) embedded within the process and the treatment of patients” [I10] “It’s not only the system integration and interoperability but also that workflow integration. So, it helps as a reminder, and it smoothens out the process itself” [C^b^14]
	Collaboration and transparency (n=12)	“For the work on the interdisciplinary team is—it has very good impact. Because, we are working interdisciplinary with surgical dermatologists. And of course, not every time is the surgical physician here; but with the app, we have the possibility here to make a picture and call him” [C11] “The advantages lie in the improvement of the interdisciplinary cooperation of different medical disciplines and the closer link between inpatient and outpatient treatment pathways” [C18]
	Clinical practice and infrastructure (n=8)	“And in some of the hospitals, it’s as well the lacking of mobile devices readiness or how to deal with mobile devices, etc. So, it’s more an infrastructure or strategic issue there” [P^c^1]
	Ease of work (n=6)	“It’s making the work a lot easier for us” [C11] “it’s easier for the physician to see something in a picture than to read it out of some long description someone did before” [I12]
	Workload and resources (n=5)	“Before taking the decision to adopt we have to check the needed infrastructure for the app. Do we have the technology to roll it out and to use it? And how much work or support does it need to keep on going?” [I12] “Digitalization is an aid, but it is currently exacerbating the speed and increasing the challenges to performance. It set a much bigger pressure on working forces by creating more demands and increasing speed of everything” [C17]
	Empowerment (n=3)	“You have the power of data so it’s a gift in who has the knowledge and often it is used by physician. Physician has the knowledge, has the information in his folder and is coordinating everything, and it gives him big power” [CP8]
	Roles and responsibilities (n=1)	“our professional patient photographer is consulted less frequently, this has changed...it (the app introduction) altered the role of the photographer, it diminished the role a little bit” [C5]
**Inner setting**		
	Decision maker (n=14)	“decision as it needs quite an intense integration and partnership it will be the IT that makes the decision. But the one that push the decision and that make this decision come through and that is behind the product is really the health care professional” [CP8] “It took us ages to get through with it. But that was an organizational problem...we had no IT personnel; we had the missing responsibility...we needed buy-in from the local IT guys. And we also need the buy-in from the local MDs of the hospitals, or the managing directors of the hospitals, and so on” [I13] “It’s also one of the barriers, I think. I mean, I’m not totally sure if it was a decision of the ICT department of the medical service...I think it was in connection between the mobility project and the ICT department” [C14] “I think the problem is nobody’s actually willing to make a decision. Everybody wants it. Everybody thinks this is great. But nobody actually says ‘Yes. This is going to be implemented’” [P15]
	Apps replacing traditional tools (n=10)	“we obviously wanted to reach more efficiency of daily clinical work because before sometimes you had to find one of the digicams, and they were not that frequent. And it had to be charged, and we needed an SD card. Later on, the SD card had to be brought somewhere else, and he had to store it in an old-fashioned folder system (laughter). So, more efficiency, higher satisfaction for the health care professional itself by more comprehensive documentation” [C5] “So, the main aspect, the main benefit, is that the manual process that was previously used, I mean, using a point-and-shoot camera and having to transfer the photos from the camera to the computer and then saving them to the right patient. This whole manual process is, yeah, completely replaced by the automatic process. So, it is a lot of time-savings and quality improvements because of the no errors, manual errors, linking the wrong photo to a patient or not linking them at all” [P15]
	Training and education (n=4)	“And when you have high fluctuation of personnel, then you have the problems. You always have to do the training” [C5]
	Innovation and tension for change (n=3)	“And I think competition with other health care providers is a topic” [C5] “the fact we use such an app can also be used in communication, that is something that we use as a tool to also kick off the internal change process in the people and show that (our institution) is an enormous player and open to that kind of innovation” [I13]
	Trialability and piloting (n=2)	“One of the factors is simply pilot projects are available and recommended to take away the fear that something goes wrong” [P7]
**Patient-related themes**		
	Patient engagement and safety (n=11)	“When you do the things manually at the end of the evening, there’s a risk that some picture from patient A go to patient B with the wrong metadata like...I can see it on the picture but is this the right leg or I don’t remember and so on. It looks like the left leg. With the app, you do it straight and it’s finished and you can work on something else. And the safety and the time is really big thing” [CP8] “And it’s much safer because you have the documentation and you can see it the next time. So, you can compare it with each other” [C11] “And the cameras, it was always difficulty because you had to go with the SD card to the computer, load it up to the right patient, and the pictures in the SD card, they are not organized. They are just a number, and if you are not watching correctly, you’re doing easy mistakes. And in imito, you are more protected from doing these kinds of mistakes” [I12] “Especially in wound care, they often adapt a treatment because a treatment is not necessarily working. And when they have the photos on the smartphone, they can easily talk to the patient and show them that they can be involved much, much more easily than before because everything is available” [P15]
	Accessibility and availability (n=2)	“The course of healing can be determined by means of photo documentation and information exchange with, eg, outpatient wound care providers and care facilities. For this, the patient does not necessarily have to be presented in the hospital or specialized facility. Unnecessary and long transport routes for patients, eg, from nursing homes are often preventable” [C18]
User engagement (n=8)		“And then the second is that they realize we’re not coming with a solution that we have to onboard the hospital, we do it reverse, we onboard into the hospitals, so they normally stay calm when they realize, aha, you come into our information system, and you work so long until your app works in our system” [P7] “One of the main parts is the users—so if we have something we think about we could use, we going to show it to the end users and they are pretty much deciding if, in first case, do they actually want it, or do they need it, or they don’t” [I12] “The first thing is that we develop our apps, not on our own. We develop them with the customer. And this really helps to create an app that is made by the customer and for the customer. And then we do a lot of feedback rounds...we go to the customers, to the users, and ask for their feedback and we prioritize” [P15]
**Policy and regulations**		
	Reimbursement and funding (n=4)	“And there will be no compensation, currently, at least. There will be no compensation for digital solutions, since the federal states are not paying for that...We are working on that. So, we are in close contact with a couple of institutions in the government in order to find some kind of compensation for that kind of expenses” [I13]
	Regulations (n=3)	“...you need a lot of resources and a lot of knowledge to develop a health app. But it’s also not so easy to get it through approval, there’s a lot of regulations” [C14] “The limitations and problems are rather in the legal area, as the sending of sensitive patient data is very restrictive...Legal and technical requirements for secure data transfer must be dealt with” [C18]

^a^I: informatics.

^b^P: provider.

^c^C: clinician.

## Discussion

### Understanding the App’s Utilities and Limitations

Participants found the app generally useful, with most users using all the key features and the main utility being efficacy and time-saving as taking clinical photos and documenting them using the app is much quicker and easier. This matches the findings from previous studies that suggested that anticipated improved efficacy enhances the intention to use [2,32-38]. Another utility is the improved patient safety and quality of care by structurally showing each case’s progression and enabling the care team to optimize the treatment accordingly; this too validates previous findings that showed mHealth may enable early detection and documentation, resulting in greater safety for patients [39-48].

Clinicians and medical informatics experts were very appreciative of the security and validation aspects of the app, stressing the importance of GDPR compliance and patient data privacy. This utility is of great importance given the typical medicolegal concerns related to confidentiality, inappropriate use, and anonymity of health data [12,45,49-72]. The accessibility and compact overview were clearly an added value of the app as they facilitated timeliness and collaboration, which were also reported in other studies that highlighted how the portability of mHealth tools enabled the care teams to easily access information and flexibly perform tasks anytime and anywhere [1,2,73-76].

Several limitations mentioned by the participants were also reported in previous studies about clinicians’ adoption of mHealth, such as potential data quality and management issues [77,78], potential information overload [79-81], and challenges related to data integration and exchange [82-85]. As for the features that the participants wanted added to the app, it was noteworthy to see that some current features such as integration with radiology devices and the offline functionality were on the wish list, revealing that users were not always aware of all available functionalities and underlining the vital role of training and education. Some of the other requested features such as the patient interface are already under consideration by the imito team but are sometimes stalled because of their high development cost.

### Understanding Constraints and Affordances

When exploring the factors affecting adoption, users reported several constraints and affordances because of not only technical functionality but also app utility in relation to the particular social and individual context of use. Usefulness was the most prominent technical factor relating to the app’s features. In alignment with previous research, perceived usefulness was closely related to time-saving and efficacy resulting from the usage of the app [2,47,48,86], its positive impact on the quality of patient care [66,72,87,88], and the potential benefit for research and scientific evidence because of better data availability [42,89]. Perceived ease of use is an equally important facilitator that has been widely reported in similar studies [2,90-93].

Participants also emphasized the importance of IT factors such as the interoperability and integration of the app with the local system in the hospital or clinic, as this would help them avoid the extra work of having to enter the same data again in the system and what it might entail from documentation errors. This has been perceived as a strong advantage of imito. Interoperability is a known challenge for mHealth and has been reported in many other studies [58,59,94-97]. These are system-level integration issues requiring that the app function properly in relation to existing systems and not just within the bounds of a given mobile device, in addition to other technical issues such as log-in or poor connectivity, which may hinder adoption [64,98-101].

Given the highly regulated nature of health care, factors such as patient data privacy and security are vital for adoption. In the case of imitoCam, this factor was perceived as a facilitator, as the app offers a secure solution for clinical photo documentation, whereas other studies reported this as a barrier if data privacy and security could not be guaranteed [53,59,66,68,82,85,102]. Security requirements thus boost the adoptability of apps that can meet stringent requirements in this regard. Another common challenge relating to data is their management and interpretation, especially when the ease of use of such apps increases the data captured and generated significantly, as discussed in other studies [40,77,78,81,84].

Of course, the cost and cost-benefit play a key role. These may be perceived as facilitators when the app helps in saving costs by creating efficiencies as narrated in similar studies [67,83,103,104]; however, they may also be a barrier [56,105,106] considering the tool’s direct costs and the indirect costs related to creating a suitable infrastructure, such as providing handheld devices across the clinic or hospital to support the app’s usage.

The findings also revealed that adoption decisions rely not only on the technical and material factors such as app features and the available infrastructure but also embrace some important social and cultural aspects. For instance, the users’ individual characteristics such as their previous experience with technology generally, and mHealth specifically, may influence their decision to adopt, as reported by other researchers [1,58,101,107,108]. Their attitudes (eg, resistance to change, risk aversion) may also hinder adoption, and comparable findings were described in earlier studies [39,40,82,109,110]. Even though mHealth is no longer a new concept, cultural views on the use of mobile devices at work may be a barrier [2,111-113]; this is slowly changing and people are accepting these tools more as per our study participants.

### Understanding How Technology Materializes in the Organizing Process

The organizational and policy implications of the app’s usage were quite prominent, showing that the interaction between the users and the technology creates the adoption patterns that we observe and influences the way people organize their work when using these new tools.

The app’s introduction created workflow advantages by offering location flexibility and a better workflow fit as data could be accessed at the point of care and easily embedded in the patient’s treatment process—a finding that is aligned with the findings of other studies that a good workflow fit encourages adoption [33,68,91]. Making daily work easier and improving collaboration and transparency were additional workflow advantages described by many participants, as they observed that the app made interdisciplinary teamwork easier and provided more transparency as a direct result of better documentation, which is also in line with the findings of other studies regarding the impact of mHealth on cooperation [12,33,40,67,69,91,103] and streamlining clinical work [114-116]. Empowerment resulting from data availability is another advantage as the app instantly equips clinicians with all the information they need, helping them make more informed decisions, as reported in similar studies [2,100]. Conversely, some other studies stated that mHealth might be perceived as a threat to clinicians’ autonomy [43,80,117,118]. We did not find this to be a concern among the participants of this study.

However, workflow disadvantages were apparent around workload and resources. Most clinical staff are already overstretched and the lack of resources may consequently be a barrier to adoption, which has been similarly described in other studies [12,39,92,119,120]. In addition, as one of the participants explained, the greater efficiencies resulting from an app such as imitoCam may also result in a higher workload that could be perceived as an additional burden for clinical staff. The implementation of these new tools may also result in changes in the roles and responsibilities of staff members as reported in other research [45,97,121,122], such as decreasing or eliminating the role of professional photographers as the app replaces their role. Furthermore, the lack of preexisting use of mobile devices in traditional clinical practice is also a challenge as it requires not only workflow adaptations but also infrastructure changes on the part of the hospital.

The nature of the organization of the hospital or clinical setting is also vital for the successful adoption and implementation of mHealth. Factors such as an ambiguous or complicated decision-making process may delay the adoption decision or even prevent it. Other researchers have also shed light on this issue [62,81,92,123], and many participants pinpointed that it is not always easy to identify the people that should be involved in deciding about a new mHealth tool in their organization, and even when they are identified, the process can be quite challenging as the decision involves an interdisciplinary team spanning IT, medical informatics, finance, and medical staff. Given this complexity in decision making, factors such as the trialability of the app and the possibility of piloting it may encourage adoption as it enables decision makers to try a new tool without risking the failure of a broad rollout [48].

Training is another vital aspect of app adoption. This is especially important when the turnover of personnel is high. This point confirms other research that has highlighted the importance of training and education [94,95,124-126]. The organization’s desire to be perceived as innovative may also facilitate the adoption of new health apps, a finding that is aligned with those of other studies showing that institutional innovation and openness to change may play a role in the adoption decision [48,127,128].

Patient-related factors reflecting the implications of app usage on quality of care also have an impact on clinicians’ adoption of mHealth. Apps that enhance patient engagement and safety, such as imito, have better chances of being adopted. This has also been described in other studies [89,102,129,130]. Similarly, there is a higher acceptance of apps that improve patient access to care [39,67,103]. Furthermore, the external context of the organization, including policy and regulations, may impact adoption. Participants explained that more clarity and simplification of the relevant regulation may facilitate adoption. Specific regulations regarding reimbursement and funding are of special importance for mHealth adoption [52,66,67,84,94,105,131]. The lack or ambiguity of such regulations may hinder the compensation of clinical activities performed via mHealth and may accordingly discourage adoption.

User engagement in the development and implementation of the app is vital for its success [48,54,69,71,99,132-134], and our findings show that this is a mutual responsibility of the health care organization and the mHealth provider. imito offers a best practice example of embedding the partners on the hospital or clinic’s side in the development and implementation processes. In this case, they constantly work together with the partner organization (hospital or clinic) in a one-team approach to ensure that the app fits into the technical infrastructure (interoperability and integration) and clinical workflow, taking into account optimization of the clinicians’ daily work through the app. This was emphasized in the functionalities implemented to ensure integration with the hospital’s systems and processes such as using patient barcodes to identify patients, directly documenting photos in the EMR, and enabling clinicians to log on to the app using the personal code on their hospital badge.

Incorporating user feedback and input in the continuous development and testing of the app is not without challenges, especially when the tool is completely embedded in the hospital’s mostly closed system, which results in blocking the visibility of usability statistics from the tool provider. However, imito has established various feedback channels where they get the opinions of the users as well as the inputs of the medical informatics experts that run the tool on the hospital’s side to ensure that their app remains relevant and useful.

### Limitations and Recommendations for Future Research

This qualitative case study has some limitations that we would like to outline. Our study is limited to a particular mHealth tool and 2 countries in a specific timeframe, and generalization to other settings that might have different characteristics, such as a different regulatory landscape, is not possible. Moreover, the relatively small sample size and the dynamic nature of mHealth necessitate a constant update of the findings to cope with the changes. The sample also excluded nonusers as their recruitment proved to be very challenging, and there is a possible favorable bias in the subgroup of participants who work for the technology provider. Future research may address some of the cited limitations by covering other apps in other countries, timeframes, and settings.

### Conclusions

This study demonstrates the utility of the studied app for clinical photo and wound documentation. The app’s adoption resulted in several benefits from the participants’ perspective, such as time-saving and efficacy, better patient safety and quality of care, data security and validation, and better accessibility. Technical and material factors affecting adoption are usefulness, ease of use, interoperability, cost, and security of the app, whereas social and cultural factors include personal experience, awareness, and attitudes.

Workflow advantages resulting from the app’s adoption include better collaboration and transparency, streamlined daily work, clinician empowerment, and improved quality of patient care. Although workflow disadvantages are associated with the available clinical practice infrastructure, workload and resources, the complexity of decision making in hospitals, the need for continuous training, and the lack or ambiguity of regulations, active user engagement in the development and implementation process may help overcome some of the workflow challenges.

A deeper look into the factors that afford or constrain user acceptance helps us understand materiality at the intersection of social and technical aspects, as the findings show that mHealth adoption is affected not only by the technical features of the app itself but also other social and organizational factors that come into play. These relationships among the technical, social, and organizational factors demonstrate that a successful acceptance and implementation of medical apps not only relies on the tool itself but also necessitates a close collaboration among the tools’ providers, clinicians, and decision makers, so that they can address the barriers and harness the potential of these new tools in advancing health care.

## Appendix

Multimedia Appendix 1 Visual of imitoCam app.

Multimedia Appendix 2 Key features of imitoCam at the time of writing this paper.

Multimedia Appendix 3 Integration of imitoCam into hospital information systems.

Multimedia Appendix 4 Interview guide.

Multimedia Appendix 5 Phases of thematic analysis.

Multimedia Appendix 6 Researcher-driven versus data-driven coding scheme.

## Abbreviations

EMR: electronic medical record
GDPR: General Data Protection Regulation
IT: information technology
mHealth: mobile health
QR: Quick Response
