# Supplementary material for: Factors Impacting Clinicians’ Adoption of a Clinical Photo Documentation App and its Implications for Clinical Workflows and Quality of Care: Qualitative Case Study
Source: JMIR Mhealth Uhealth. 2020 Sep 23;8(9):e20203. doi: 10.2196/20203 (PMC7542402; doi:10.2196/20203)
Supplement: Multimedia Appendix 1 [file mhealth_v8i9e20203_app1.pdf]

imitoCam - Documentation Process

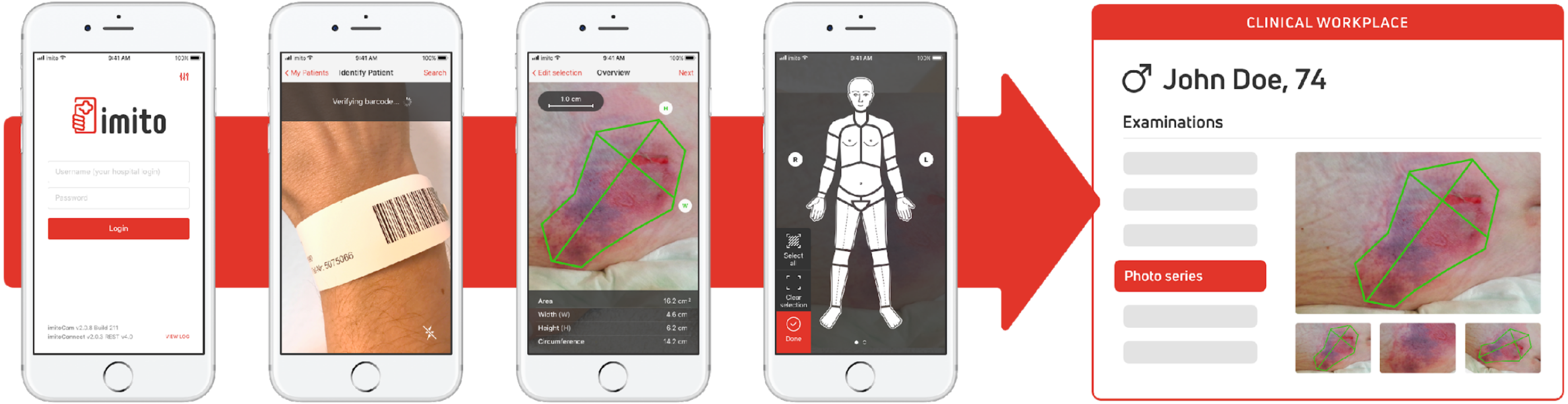

With imitoCam, the clinical photo-documentation is seamlessly embedded in the clinical documentation process.

1. The user logs in with his Active Directory login and identifies the patient via barcode.
2. In the patient context, previously taken photos and videos are available in a timeline.
3. Photos, videos, and wound measurements are documented.
4. Findings are categorised, transferred to the patient electronic medical record and archived.
5. Documented findings are available through the clinical workplace.

imitoCam - Optimised Workflow

Immediately in the context

Hassle-free user login via barcode/RFID for impersonal devices and direct patient identification via barcode.

Offline and emergency mode

Capture series offline or without patient identification and complete it later on any device.

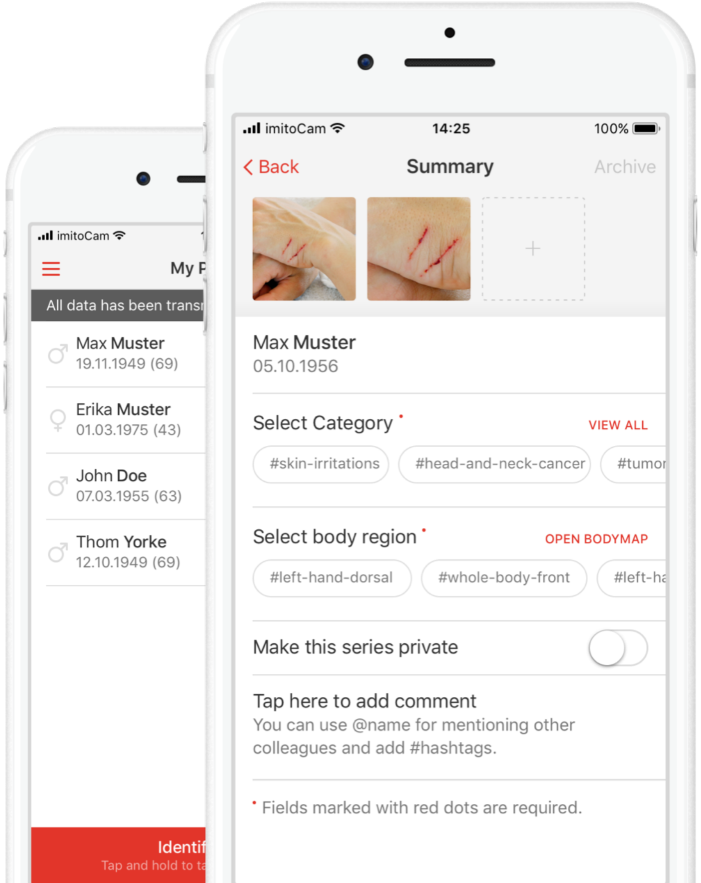

Order-based

An order-processing-interface allows seamlessly integrated wound photography workflows based on tasks.

Optimised usability

A new workflow enables a quick and less error-prone documentation.

imitoCam - Wound Measurement

Wound measurement

Automatic and precise measurement of the area, length, width and circumference of wounds and specimens.

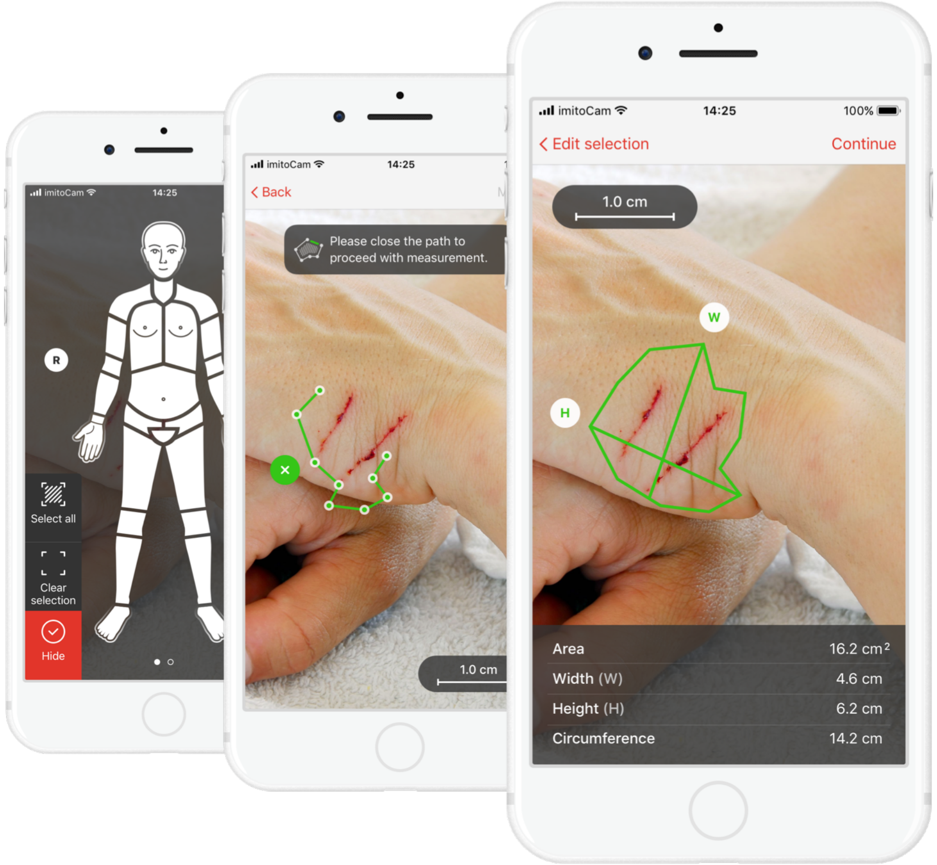

imitoCam - Patient Timeline

Timeline

Browse through the timeline of one or more findings of a patient and better understand the progression.

Interdisciplinary communication

Enables secure and convenient team collaboration via chat, e.g. for second opinions.

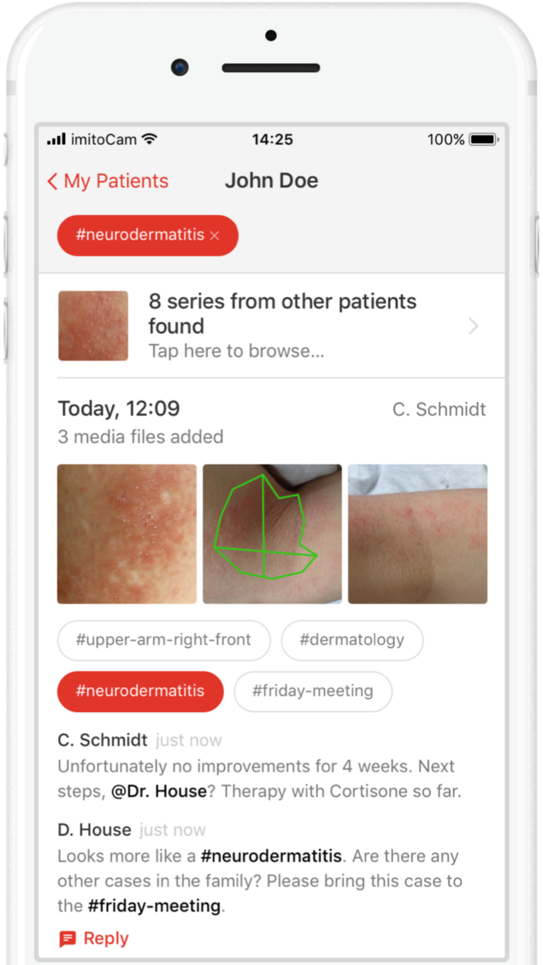

Intelligent Search

Find other photos with similar characteristics.

Hashtags

The smart and dynamic categorisation of images provides added-value for research and education.
