# Supplementary material for: Factors Impacting Clinicians’ Adoption of a Clinical Photo Documentation App and its Implications for Clinical Workflows and Quality of Care: Qualitative Case Study
Source: JMIR Mhealth Uhealth. 2020 Sep 23;8(9):e20203. doi: 10.2196/20203 (PMC7542402; doi:10.2196/20203)
Supplement: Multimedia Appendix 2 [file mhealth_v8i9e20203_app2.pdf]

imitoCam - Key features

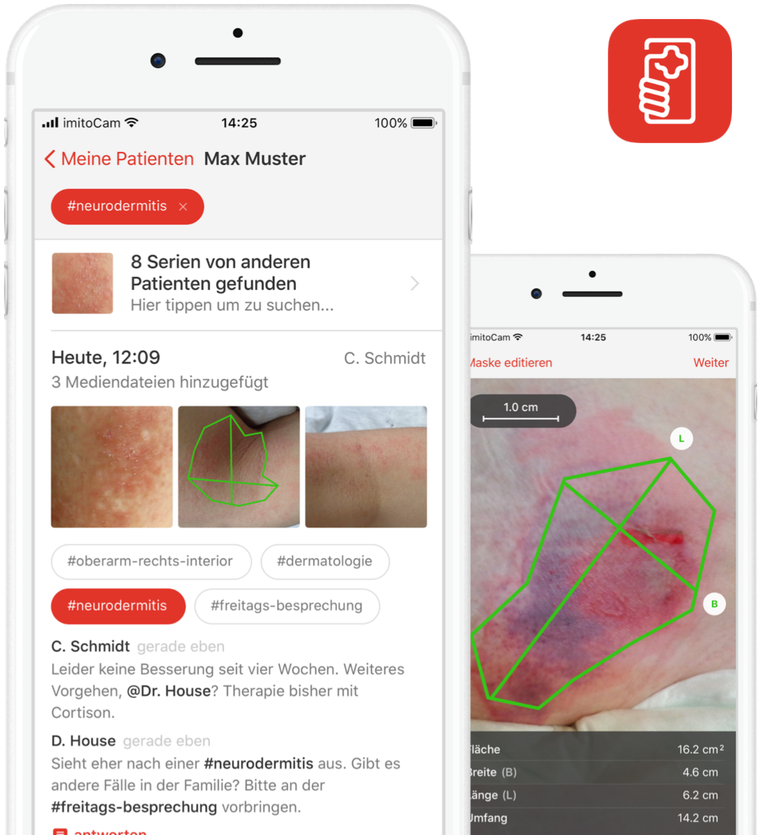

- Immediately in the **patient context**
- Timeline for **effective data presentation**
- Chat for **team collaboration** & second opinions
- Precise **wound measurement**
- **Seamless** and standardised **integration**

imitoCam - Data Protection

VIP Patients

Patients with VIP status (hospital employees for example) do not appear in the patient search index.

Encrypted

Hardware encryption of temporary data (AES-256), secure transmission to server (TLS).

Secure unlocking

Easily unlock imitoCam during the hectic daily routine: with TouchID/FaceID or PIN on personal devices, with employee ID card (NFC) on shared devices.

Sensitive content

Identify image series with sensitive content, e.g. victims of violence, so that they are only visible to the author.

No-Footsteps

After the end of a user session, all personal data is removed from the mobile device.

No cloud

The data belongs to you and remains on your infrastructure.
