# Supplementary figures and images for: Factors Impacting Clinicians’ Adoption of a Clinical Photo Documentation App and its Implications for Clinical Workflows and Quality of Care: Qualitative Case Study
Source: JMIR Mhealth Uhealth. 2020 Sep 23;8(9):e20203. doi: 10.2196/20203 (PMC7542402; doi:10.2196/20203)

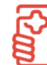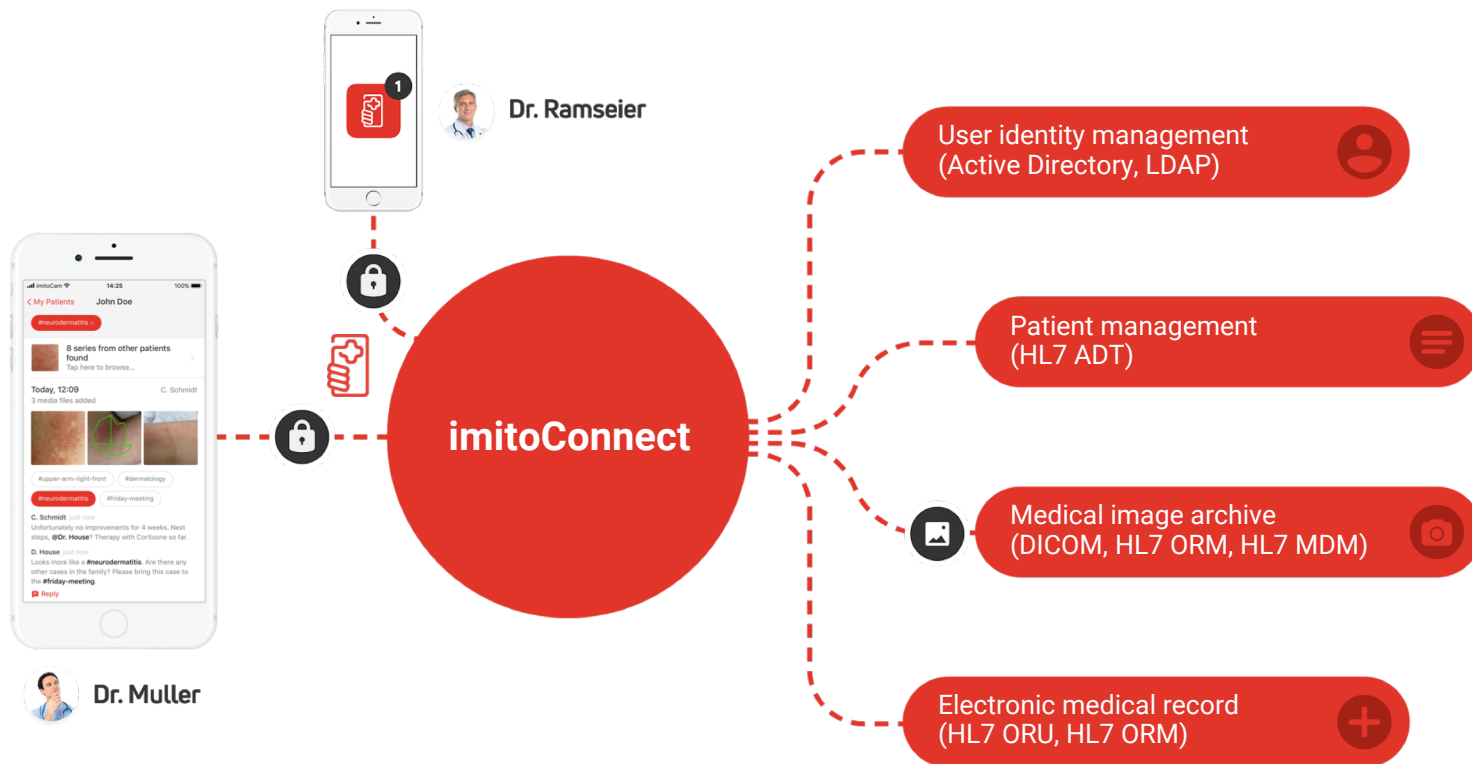

Supplement: Multimedia Appendix 3 [file mhealth_v8i9e20203_app3.pdf]
