# Supplementary material for: Factors Impacting Clinicians’ Adoption of a Clinical Photo Documentation App and its Implications for Clinical Workflows and Quality of Care: Qualitative Case Study
Source: JMIR Mhealth Uhealth. 2020 Sep 23;8(9):e20203. doi: 10.2196/20203 (PMC7542402; doi:10.2196/20203)
Supplement: Multimedia Appendix 4 [file mhealth_v8i9e20203_app4.pdf]

Interview guide - *Clinicians*

Background Questions

1. Participant introduction
   - Tell me about your role in the organization
   - How long have you worked in healthcare?
   - How long have you been using Mobile Health?
   - How would you define your level of technical awareness on a scale of 1 to 10?

2. How would you define "Mobile Health" in one sentence?

Theme 1: "Accounting for materials"

3. Tell me about the Imito app
   - What are its main features?
   - Are there any limitations in its features?
   - If you would add one feature what would it be?

4. How did it help you and your patients?

Theme 2: "Accounting for materiality"

5. Tell me about what you wanted to achieve when you decided to use the Imito app

6. What were the factors that influenced your decision to adopt the Imito app?
   - Which would you consider a barrier and which an opportunity?

7. Who made the decision to implement the Imito app? And are there in assessment/selection criteria for such new technologies in your workplace?

Theme 3: "Accounting for materialization"

8. What influence did the Imito app have on your work/the work of others (e.g Workflow)?
   - Did it improve it?
   - Was the previous practice better for some things?

9. Have these solutions led to changes in how the organization works, its rules or the use of other tools / technologies?

10. How have the uses of the Imito app sustained, altered, or transformed the way that people interact in your organization?

11. In your opinion, what does the future hold for mHealth? And what roles will HCPs play in shaping this future?
