# Supplementary material for: Factors Impacting Clinicians’ Adoption of a Clinical Photo Documentation App and its Implications for Clinical Workflows and Quality of Care: Qualitative Case Study
Source: JMIR Mhealth Uhealth. 2020 Sep 23;8(9):e20203. doi: 10.2196/20203 (PMC7542402; doi:10.2196/20203)
Supplement: Multimedia Appendix 6 [file mhealth_v8i9e20203_app6.pdf]

Appendix 6: Researcher-driven versus data-driven coding scheme

Theme	**Sub-theme**	**Researcher/ Data Driven**	**Theoretical frameworks where the researcher driven themes originated**
Technical and material factors			
Data Related	Data management	Data driven	__
	Privacy, security, and liability	Data driven	__
Ease of Use		Researcher driven	Perceived ease of Use (TAM)-Effort expectancy (UTAUT)-Complexity (CFIR-DOI)
IT Capability and compatibility	Interoperability and integration	Data driven	__
	Technical issues	Data driven	__
Monetary factors		Researcher driven	Cost (CFIR)-Affordability (APPEASE)
Usefulness	Efficacy and time saving	Researcher driven	Effectiveness (APPEASE)
	Evidence base	Researcher driven	Evidence (CFIR)
	Quality of care	Researcher driven	Output quality (TAM2)
	Usefulness	Researcher driven	Perceived usefulness (TAM)-Performance Expectancy (UTAUT)-Relative Advantage (CFIR-DOI)
Social and individual factors			
Personal characteristics	Attitude	Researcher driven	Attitude (TAM-TPB)
	Awareness	Data driven	__
	Experience and habits	Researcher driven	Self-Efficacy (CFIR)- Habit (TIB)
Social and cultural factors	Culture	Researcher driven	Culture (CFIR)
Organizational and policy implications			
Inner setting	Apps replacing traditional tools	Data driven	__
	Decision maker	Data driven	__
	Innovation and tension for change	Researcher driven	Tension for change (CFIR)
	Training and education	Data driven	__
	Trialability/piloting	Researcher driven	Trialability (CFIR)
Workflow related	Clinical practice and infrastructure	Researcher driven	Compatibility (CFIR-DOI)-Adaptability (CFIR)-Practicability (APPEASE)-Job Relevance (TAM2)
	Collaboration and transparency	Data driven	__
	Empowerment	Data driven	__
	Ease of work	Data driven	__

	Roles and responsibilities	Data driven	—
	Workflow fit and location flexibility	Data driven	—
	Workload and resources	Researcher driven	Resources (CFIR)
Policy and regulations	Regulations	Researcher driven	External Policies (CFIR)
	Reimbursement and funding	Data driven	—
Patient related	Accessibility and availability	Data driven	—
	Patient engagement and safety	Data driven	—
User engagement		Data driven	—
